# Temporal and Molecular Analyses of Cardiac Extracellular Matrix Remodeling following Pressure Overload in Adiponectin Deficient Mice

**DOI:** 10.1371/journal.pone.0121049

**Published:** 2015-04-24

**Authors:** Keith Dadson, Subat Turdi, Stellar Boo, Boris Hinz, Gary Sweeney

**Affiliations:** 1 Department of Biology, York University, Toronto, Canada; 2 Laboratory of Tissue Repair and Regeneration, Matrix Dynamics Group, Faculty of Dentistry, University of Toronto, Toronto, Canada; Albert Einstein College of Medicine, UNITED STATES

## Abstract

Adiponectin, circulating levels of which are reduced in obesity and diabetes, mediates cardiac extracellular matrix (ECM) remodeling in response to pressure overload (PO). Here, we performed a detailed temporal analysis of progressive cardiac ECM remodelling in adiponectin knockout (AdKO) and wild-type (WT) mice at 3 days and 1, 2, 3 and 4 weeks following the induction of mild PO via minimally invasive transverse aortic banding. We first observed that myocardial adiponectin gene expression was reduced after 4 weeks of PO, whereas increased adiponectin levels were detected in cardiac homogenates at this time despite decreased circulating levels of adiponectin. Scanning electron microscopy and Masson’s trichrome staining showed collagen accumulation increased in response to 2 and 4 weeks of PO in WT mice, while fibrosis in AdKO mice was notably absent after 2 weeks but highly apparent after 4 weeks of PO. Time and intensity of fibroblast appearance after PO was not significantly different between AdKO and WT animals. Gene array analysis indicated that MMP2, TIMP2, collagen 1α1 and collagen 1α3 were induced after 2 weeks of PO in WT but not AdKO mice. After 4 weeks MMP8 was induced in both genotypes, MMP9 only in WT mice and MMP1α only in AdKO mice. Direct stimulation of primary cardiac fibroblasts with adiponectin induced a transient increase in total collagen detected by picrosirius red staining and collagen III levels synthesis, as well as enhanced MMP2 activity detected via gelatin zymography. Adiponectin also enhanced fibroblast migration and attenuated angiotensin-II induced differentiation to a myofibroblast phenotype. In conclusion, these data indicate that increased myocardial bioavailability of adiponectin mediates ECM remodeling following PO and that adiponectin deficiency delays these effects.

## Introduction

The physiological significance of myocardial extracellular matrix (ECM) regulation by adipokines in diabetes and obesity has been well established [1–3]. In particular, regulation of cardiac remodelling by adiponectin is thought to be of great significance [3]. Adiponectin is present at high levels of 2–20 ug/ml in the circulation of normal individuals but levels are reduced in diabetes and obesity [3,4]. The discovery of adiponectin production in the heart has led to the hypothesis that adiponectin acts as a cardiokine [5]. Early studies showed that adiponectin deficiency in mice exacerbated stress induced cardiac remodelling and failure [6–8]. Recently however, both clinical and animal studies have also suggested that adiponectin supports remodelling events leading to heart failure [9–11]. Meta-analysis of clinical studies correlating adiponectin with various heart disease outcomes has been inconclusive so far [12–14]. Clearly, more insight into the effects of adiponectin and mechanisms of action in the pathogenesis of pressure overload (PO) induced cardiac remodelling is needed.

Cardiac ECM remodelling plays a critical role in the adaptation to haemodynamic stressors and excessive remodeling ultimately results in the progression to heart failure [2]. In mouse models, PO is commonly associated with enhanced collagen deposition in the heart. The transition from compensatory ECM support to detrimental fibrotic stiffening occurs when hypersecretory myofibroblasts predominate in the overloaded myocardium [15]. Myofibroblasts are contractile fibroblastic cells that are characterized by actin/myosin bundles (stress fibers) that express α-smooth muscle actin (α-SMA) în their fully differentiated state [16]. Remodelling of the myocardial collagen ECM is primarily mediated by matrix metalloproteinases (MMPs) [17] and their role in heart failure is now well established [1,18]. During the process of remodelling, MMPs are initially activated to reduce wall stress by increasing fibrillar collagen degradation, allowing left ventricle (LV) dilation in response to increased work load. Ultimately, prolonged MMP activation adversely affects cardiac function since the ultrastructural fibrillar collagen which is initially degraded by MMPs is replaced by poorly structured collagen [19]. Changes in MMP levels have been described in both human and a variety of animal models of heart failure [18,19]. Chemical inhibition of MMPs has also been shown to attenuate LV dilation and preserve function after surgical induction of infarction [20]. Little is known regarding the effects of adiponectin on myocardial MMP activity in models of heart failure, although studies have shown direct effects of adiponectin on MMP and other ECM-related genes in primary cardiac fibroblasts and myocytes [21,22].

The aim of our study was to conduct a detailed temporal investigation of cardiac ECM remodelling after PO in WT versus adiponectin knock-out (AdKO) mice. We examined changes in collagen expression and deposition, myofibroblast content, and changes in MMP and tissue inhibitors of MMPs (TIMP) expression over time. We also investigated changes in cardiac adiponectin expression and content, and studied the direct effects of adiponectin on various end points relevant to ECM remodelling in primary cardiac fibroblast (CF) cultures.

## Materials and Methods

### Materials

Recombinant full-length adiponectin was produced in-house as previously detailed by [23]. Dulbecco’s modified eagle’s medium (DMEM), trypsin, antibiotic/antimycotic and fetal bovine serum (FBS) were obtained from Gibco Laboratories (Life Technologies Inc., Burlington, ON, CA). All culture plates were BD Falcon brand and purchased from BD Biosciences (Mississauga, ON, CA). TRIzol Reagent was purchased from Ambion Inc. (Life Technologies Inc.), and adiponectin primers were obtained from Invitrogen (Life Technologies Inc.). VECTASHIELD mounting medium with DAPI was obtained from Vector Laboratories (Burlington, ON, Canada). RNeasy MinElute Cleanup and RT^2^ First Strand kits were purchased from QIAGEN Inc. (Mississauga, ON, CA). ^3^H-proline was purchased from Amersham Biosciences (GE Healthcare Lifesciences, Baie d’Urfe, QC, CA). Amicon Ultra-15 Centrifugal Filter Units were obtained from EMD Millipore (Billerica, MA, USA). The Pierce BCA Protein Assay kit was purchased from Thermo Scientific (Fisher Canada, Nepean, ON, CA). The custom fibrosis PCR array was purchased from SABiosciences (QIAGEN Inc.). LOOK silk black braided non-absorbable 6–0 USP sutures were purchased from Harvard Apparatus (Holliston, MA, USA), titanium ligation clips and microclip ligating appliers used for minimally invasive trans-aortic banding (MTAB) were purchased from Teleflex Medical (NC, USA).

### Experimental animals

In-house bred male AdKO mice [24] at 6–8 weeks of age, and age matched C57BL6 (wt) mice, were randomly allocated to treatment groups. Animal facilities met the guidelines of the Canadian Council on Animal Care, and all protocols were approved by the Animal Care Committee of York University. Animals were acclimated a minimum of 5 d to a standard housing environment: temperature and humidity-controlled rooms (21±2°C,35–40%), with a daily 12:12h light-dark cycle (lights on at 0700) with access to regular chow diet *ad libitum*.

### Quantification of collagen diameter and vimentin density

Image J (NIH) straight line measurement tool was used to quantify mean diameter of collagen fibres viewed by scanning electron microscopy at 5k magnification. Five images were taken per mounted tissue and three tissue samples were produced per animal heart (= 15 images/animal heart). Image J was used to semi-automatically quantify content of fibroblastic cells in mouse heart tissue. After manual exclusion of vessels based on morphology and α-SMA staining, the mean grey values of the vimentin immunostaining signal was quantified over the whole remaining image area (= vimentin density). Five images were taken per stained slide and three slides were produced per animal heart (= 15 images/animal heart). Mean values and standard deviations were calculated form the values obtained for three different animals per experimental condition (n = 3).

### Induction of PO via minimally invasive transverse aortic banding

Under general anesthesia (IP Xylazine 0.15 mg/g; Ketamine 0.03 mg/g), the mouse is kept in a supine position and the fur on the ventral surface removed with a depilatory cream, and the surface sterilized with betadine. A medial cranio-caudal incision is made through the skin from the neck to the bottom of the rib cage, and the neck muscles, fat, and thyroid are retracted to expose the trachea down to the suprasternal notch. An incision is made through the suprasternal notch 2–3 mm down the rib cage. The transverse aorta is visualized under low magnification between the innominate and left common carotid arteries. A titanium micro-ligation clip is applied across the transverse aorta using banding calipers calibrated to the width of a 26g needle. The sham surgery is performed as outlined above without the placement of the ligation clip. Upon completion of the procedure, the rib cage and skin are closed with silk suture, and the mice injected with Buprenorphine (s.c. 0.05 mg/kg) and placed face down on a warming pad until they awake. No animals died of cardiac failure due to the surgical intervention. Animals were monitored continually post surgery until they could roll from supine to prone position, and were ambulatory. Following this, animals were returned to the animal facilities and monitored every 6–12 houtrs until the end of the study.

### Tissue and serum collection

Mice were weighed and euthanized using cervical dislocation. Hearts were excised and perfused briefly with KCl to arrest the heat in diastole. Hearts were then weighed and divided for further analysis. Blood samples were collected at time of death, centrifuged (10,000 RPM, 5 min, 4°C) and the serum supernatant collected to analyze adiponectin content.

### Scanning electron microscopy

Heart tissue was fixed in 2% EM grade gluteraldehyde in 0.1M sodium cacodylate buffer pH 7.3 for 1 hour at room temperature, then stored in 0.1M sodium cacodylate buffer, pH 7.3, 0.2M sucrose until further processing. Fixed samples were chemically dehydrated in hexamethylsilazine, mounted on stubs and sputter-coated (Hummer VI Au/Pd 40/60) and examined with a high-resolution scanning electron microscope (Hitachi S-520) at an accelerating voltage of 20 kV equipped with a passive image capture system (Hitachi, Quartz PCI Version 6).

### Tissue histology, immunostaining, and light microscopy

Mid-ventricular cross-sections were fixed in 10% formalin solution for 1 hour then stored in 70% ethanol at 4°C until further processing. Fixed heart tissues were dehydrated to xylene and embedded in pure paraffin wax blocks. For immunofluorescence staining sections were deparaffinized in xylene, rehydrated in ethanol, and rinsed in distilled water, as described previously [25]. Antigens were revealed by boiling the slides in sodium citrate buffer (Dako, Burlington, ON) at 95–100°C for 40 min. After cooling to room temperature, sections were rinsed in PBS, blocked with 2% goat serum/1% BSA for 40 min and stained with primary and secondary antibodies applied sequentially for 1 h at room temperature. Primary antibodies directed against α-SMA (α-SM1, Giulio Gabbiani, University of Geneva, Switzerland), desmin (M076029, Dako, Burlington, ON), collagen type I (Santa Cruz, CA, USA) and vimentin (D21H3, Cell Signals), were used. Isotype-specific secondary antibodies were Alexa488-conjugated goat anti-mouse IgG1, Alexa647-conjugated IgG2a and Alexa568-conjugated goat anti-rabbit, Alexa Fluor488-conjugated goat anti-rabbit (Molecular Probes, Life Technologies Inc.) and FITC-labelled goat anti-mouse antibody (Jackson ImmunoResearch Laboratories, Inc., West Grove, PA, USA). Micrographs were acquired using an upright Zeiss Axio Observer M35 epifluorescence microscope equipped with structured illumination (Apotome) and an Axiocam HR camera (Carl Zeiss, Jena, Germany). All images were assembled using Adobe Photoshop CS4 (Adobe Systems, San Jose, CA). Contrast and brightness were enhanced identically over all images for publication purposes.

For in-vitro α-smooth muscle actin and intracellular collagen imaging, CFs were seeded onto 25 mm coverslips and grown to ∼50% confluence in DMEM containing 10% FBS before adiponectin treatment. For in-vitro extracellular collagen imaging, CFs were initially grown as above for 1 day, and then cultured for 3 days in DMEM containing 10% goat serum prior to adiponectin treatment. Following treatment as indicated, cells were gently washed with PBS, fixed with 3% PFA for 30 min at room temperature, and then incubated with 1% glycine for 10 min at room temperature to quench PFA. After fixation, the cells were then blocked at room temperature in either 5% goat serum for 1 h (α-smooth muscle actin, intracellular collagen imaging), or 1% horse serum for 30 min (extracellular collagen imaging), followed by incubation at room temperature with rabbit anti-collagen I (1:100) or goat anti-collagen III (1:100) antibody for 1 h. Cells were then incubated at room temperature with AlexaFluor 488 goat anti-rabbit (1:1,000) or AlexaFluor 594 donkey anti-goat (1:1,000) secondary antibody respectively for 1 h, followed by a final wash with PBS, and mounting on glass microscope slides using VECTASHIELD mounting medium with DAPI. Immunofluorescent images were obtained using an Olympus BX51 confocal microscope (Olympus) with 20× and 60× objectives. Quantification of collagen secretion (pirosirius red staining) was performed as described previously [21]. Side-view reconstruction of in-vitro extracellular collagen was assembled using FLOWVIEW software version 5.0 from confocal stacks of 10–15 images visualized by sequential views (Δ X: 4, ΔY: 0, ΔZ:0, number of views: 91) into a video file (.avi) and screen captured on the z-plane.

### Isolation and culture of primary rat cardiac fibroblasts

Neonatal CFs were isolated from 3–4 day old Wistar rats as previously described [23]. CFs were used at first passage for myofibroblast differentiation experiments, or passaged twice, grown to 100% confluence (or as otherwise indicated below) and then starved with serum-free DMEM for at least 3 hours prior to treatment with recombinant full-length adiponectin (5.0 μg/ml).

### Western blot analysis

Cell culture lysis and protein sample preparation was conducted according to methods detailed by [26] and tissue homogenate preparation as detailed by us before [27]. Briefly, homogenates were obtained from powderized tissue samples lysed with buffer containing phosphatase and protease inhibitors (30 mM HEPES, pH 7.4, 2.5 mM ethylene glycol tetraacetic acid, 3 mM ethylenediaminetetraacetic acid, 70 mM KCl, 20 mM β-glycerolphos- phate, 20 mM NaF, 1 mM Na3VO4, 200 μM phenylmethylsulfonyl fluoride, 1 μM pepstatin A, 10 μM E-64, 1 μM leupeptin, and 0.1% Nonidet P-40). All samples were standardized by total protein and analyzed by SDS-PAGE. Primary anti-α-smooth muscle actin, anti-adiponectin, and anti-β-actin antibodies were used at 1:1000 dilutions, and appropriate HRP-conjugated secondary antibodies were used at 1:10,000 dilutions. Proteins were detected by chemiluminescence, quantified by densitometry using Scion Image software (Scion Corp., Frederick, MD, USA) and then normalized to β-actin protein levels as appropriate.

### RNA isolation and quantitative real-time PCR

Total RNA was isolated from powdered tissue homogenates using TRIzol Reagent according to the manufacturer’s instructions, and purified using the RNeasy MinElute Cleanup Kit to attain an A_260_/A_280_ ratio between 1.9 and 2.0. First-strand cDNA, synthesized from 1 μg RNA using the RT^2^ First Strand kit, was used in a custom PCR array comprising 96-well plates pre-coated with primers listed in Table 1. Quantitative real-time PCR was conducted using a Chromo4 Detection system (Bio-Rad Laboratories Canada Ltd., Mississauga, ON, CA) according to cycling conditions outlined by the PCR array manufacturer. Data were analysed using RT^2^ Profiler PCR Array Data Analysis software (Version 3.5; QIAGEN Inc.) and normalized to GAPDH mRNA expression. Adiponectin mRNA expression (forward: 5′-GCAGAGATGGCACTCCTGGA-3′; reverse: 5′-CCCTTCAGCTCCTGTCATTCC-3′) was analyzed by quantitative real-time PCR using DyNAmo HS SYBR Green qPCR kit (Finnzymes, Woburn, MA) with a Chromo4 Detection system and the following cycling conditions: Hot start: 95°C for 15 min; 35 cycles of: 95°C for 30 s, 65°C for 30 s, 72°C for 30 s; final extension: 72°C for 10 min.

**Table 1:** List of ECM-related genes analyzed by customized PCR array and their respective Referece Sequence Number.

Gene Symbol	Gene RefSeq #
Collagen 1 α1	NM_007742
Collagen 3 α1	NM_009930
Collagen 4 α1	NM_009931
TIMP-1	NM_011593
TIMP-2	NM_011594
TIMP-3	NM_011595
MMP-1a	NM_032006
MMP2	NM_008610
MMP8	NM_008611
MMP9	NM_013599
MMP13	NM_008607
MMP14	NM_008608
GAPDH	NM_008084
β-Actin	NM_007393
MGDC	SA_00106
PPC	SA_00103

### Wound scratch migration assay

Fibroblast migration in response to adiponectin treatment was assessed using the wound scratch assay as described previously [21]. Briefly, a sterile 1 mL pipette tip was used to scratch a straight line through CFs grown to confluence. Scratched wells were starved overnight then treated with adiponectin for times indicated. Prior to fixation, freshly scratched wells served as “fresh scratch” controls. Cells were fixed in 90% methanol then mounted using VECTASHIELD mounting medium with DAPI. Fluorescent images were obtained using an Olympus BX51 confocal microscope (Olympus, Seattle, WA, USA) with a 20 x objective, and fibroblast migration was assessed as the closure of the scratch wound in arbitrary length units using Inkscape software (www.inkscape.org).

### Matrix metalloproteinase activity analysis using gelatin zymography

Zymographic analysis of conditioned media from CFs grown in 6-well plates was performed as described previously [21]. Briefly, conditioned media was concentrated following adiponectin treatment for indicated times. Equal amounts of protein from media (25 μg) were resolved by SDS–PAGE gel containing 0.3% gelatin. Matrix metalloproteinase activity was activated by incubation for 18 h at 37°C in 1 M Tris–HCl (pH 7.6) containing 100 mM CaCl2. Gels were fixed and stained with Coomassie Blue solution. MMP2 activity was quantified by densitometric analysis of degraded areas using Scion Image software (Scion Corp.).

### 3H-proline and 3H-thymidine incorporation assays

Pro-collagen synthesis and fibroblast proliferation was assessed by measurement of cellular 3H-proline and 3H-thymidine uptake respectively as previously outlined [21]. Briefly, CFs were treated with or without adiponectin in the presence of 3H-proline or 3H-thymidine (1 μCi/ml final concentration) for the indicated times. At the end of each treatment period, cells were incubated for 30 min with ice-cold 5% trichloroacetic acid at 4°C. The resulting acid precipitate was then solubilized overnight in 0.5 ml of 0.5 N NaOH at 37°C and neutralized with 0.5 ml 0.5 N HCl per well. The radioactivity of each sample was measured in a liquid scintillation counter and corrected for total protein content using the Pierce BCA Protein Assay kit.

### Analysis of adiponectin in serum

Serum from wild-type, and adiponectin heterotype animals was analyzed for adiponectin content 2 or 4 weeks following MTAB or sham surgery by ELISA kit (Antibody Immunoassay Services, Hong Kong) following manufacturer’s instructions.

### Statistical analysis

For in-vivo experiments, data are expressed as mean values ± SEM (n), where n represents the number of animals. Two-way ANOVA was used to determine significant differences (P < 0.05) between animal groups. For in-vitro experiments, data are expressed as mean values ± SEM (n), where n = 1 represents aggregated experiments per CF isolation. Student’s *t* tests were used to determine significant differences (P < 0.05) between groups. All statistical analyses were conducted using SigmaStat 3.5 Software (Systat Software Inc., San Jose, CA, USA).

## Results and Discussion

### PO induces adiponectin retention in the myocardium

**Fig 1:**
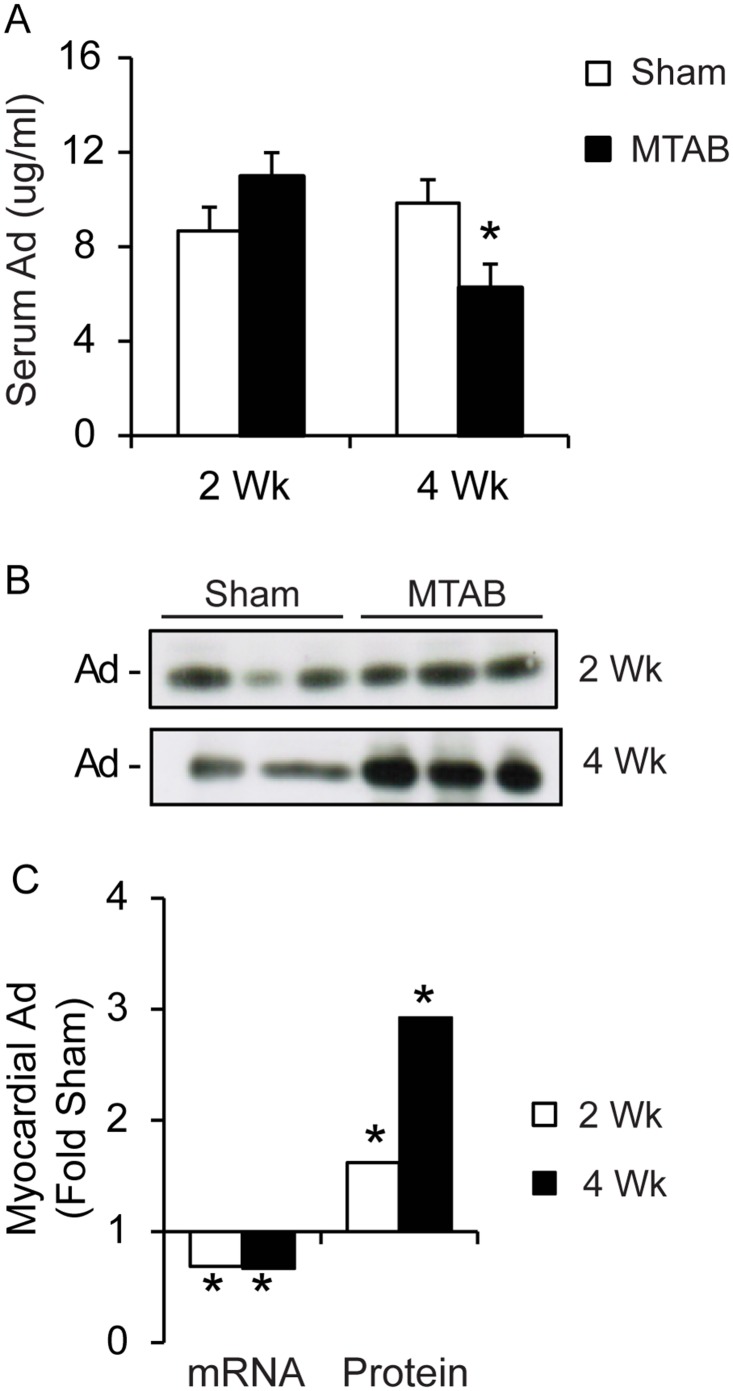
Adiponectin is retained in the heart following PO. (A) Analysis of serum adiponectin by ELISA. Serum was collected at time of euthanization from AdKO or WT mice 2 or 4 weeks following sham or MTAB surgery. Values are represented as mean of 4 to 6 mice per group ± sem. (B) Western blot analysis of cardiac homogenate samples from AdKO or WT mice 2 or 4 weeks following sham or MTAB surgery, quantified in the graph below alongside quantitative real-time PCR analysis of adiponectin mRNA obtained from cardiac homogenates isolated from AdWT mice 2 or 4 weeks following sham or MTAB surgery. Values are represented as average C(t) fold sham, where sham is set to 1. Values are average of n = 3 to 5 mice per group ± SEM.

We first examined changes in circulating and local myocardial adiponectin levels following PO. We first used ELISA to determine the amount of adiponectin in circulation of WT mice and found a small but significant decrease after 4 weeks of PO when compared to sham (Fig 1A). Interestingly, myocardial adiponectin content increased after 4 weeks of PO, with a small increase apparent after 2 weeks (Fig 1B and 1C). These changes occurred despite the fact that adiponectin mRNA levels in the heart were significantly decreased following 2 and 4 weeks of PO (Fig 1C).

### Progression of myocardial fibrosis

**Fig 2:**
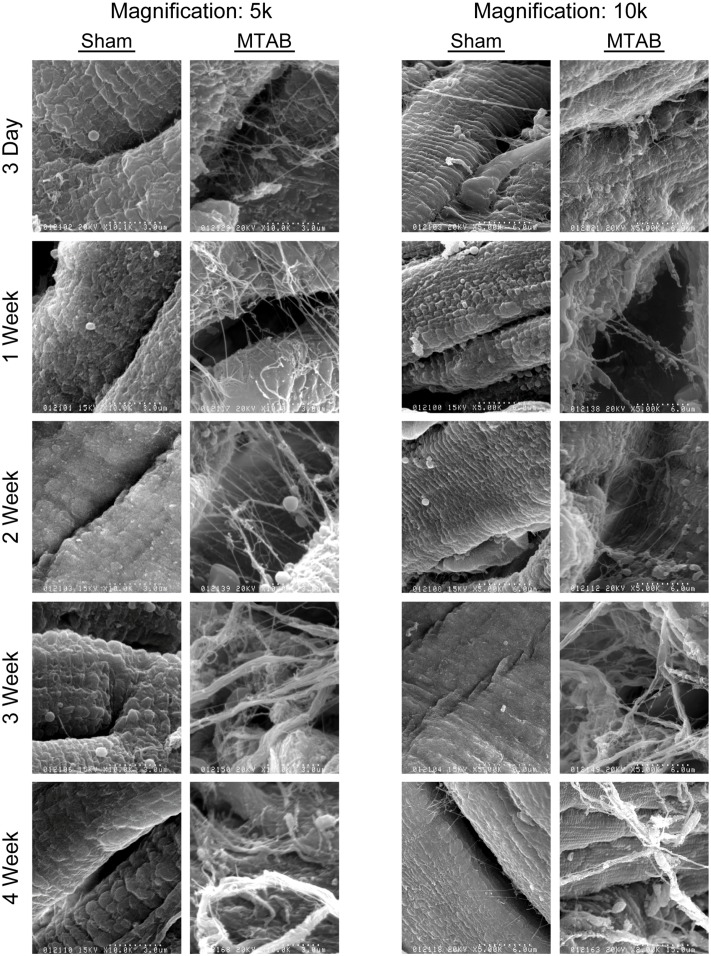
Cardiac fibrosis is temporally regulated following PO. Representative scanning electron micrographs of fixed left ventricular samples of wt C57BL/6 mice 3 days, 1, 2, 3, or 4 weeks following sham or MTAB surgery, shown at 5000X or 10000X. Images shown are representative of 5–10 images of n = 4 to 6 mice per group.

**Fig 3:**
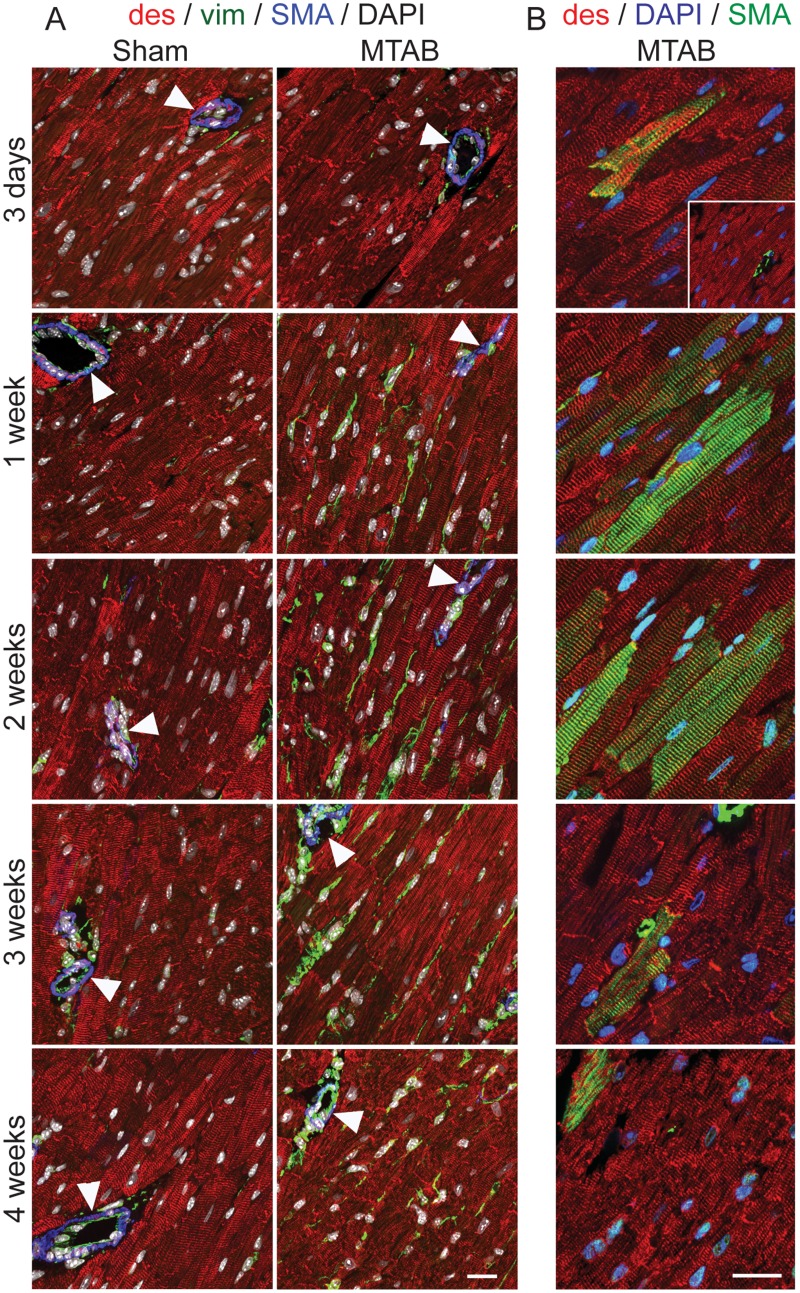
Cellular responses to PO in the myocardium. WT mice were Sham operated or subject to aortic banding (MTAB) and sacrificed after the indicated time points. Transmural blocks of the left ventricular myocardium were sectioned and immunostained for (A) α-SMA (SMA, blue), desmin (des, red), vimentin (vim, green) and nuclei (DAOI, white) or (B) α-SMA (SMA, green), desmin (des, red), and nuclei (DAOI, blue). Note that exposure time for α-SMA imaging was adjusted in (A) to the high staining intensity in vascular smooth muscle and in (B) to the comparably lower staining intensity in cardiomyocytes overloaded myocardium (~2-times longer exposure). Scale bars: 50 μm.

Accumulation of total collagen following the induction of PO has been well characterized using methods for determining total collagen content, such as Masson’s Trichrome stain [28,29]. In this study, we used scanning electron microscopy of the collagen ECM following induction of PO and observed a time dependent difference in collagen fibre size and structure (Fig 2). The myocardium in the acute period following MTAB surgery (3 d–2 weeks) exhibited an expanded network of small collagen fibres (Fig 2). Disorganized large ECM fibres structurally similar to collagen bundles, were observed later after 3 and 4 weeks of PO (Fig 2). No apparent changes were observed over time in sham operated mice (Fig 2). Increase of collagen fiber quantity and density correlated with the gradual increase of vimentin-positive interstitial fibroblasts between desmin-positive cardiomyocytes during the first 2 weeks after MTAB (Fig 3A). After peak appearance at 2 weeks, fibroblast levels stagnated at 3 weeks and decreased after 4 weeks to the level observed after 1 week; no changes over time were observed in fibroblast appearance in the myocardium of sham-operated animals (Fig 3A). In our model of mild aortic banding-induced pressure overload, the late myofibroblast marker α-SMA was never expressed in fibroblastic cells; however, neo-expression of α-SMA increased dramatically in the sarcomeres of individual desmin-positive cardiomyocytes until 2 weeks post-MTAB and decreased thereafter to the baseline levels observed in sham-operated animals (Fig 3B). As internal control for specificity of α-SMA staining served smooth muscle cells of the vasculature that also stained positive for desmin but were distinct in morphology and exhibited no sarcomere banding (Fig 3, arrowheads). The levels of α-SMA staining in vascular smooth muscle was considerably higher than in cardiomyocytes.

### Adiponectin deficiency delays the myocardial fibrotic response to PO and influences changes in expression of collagen, MMP and TIMP isoforms

**Fig 4:**
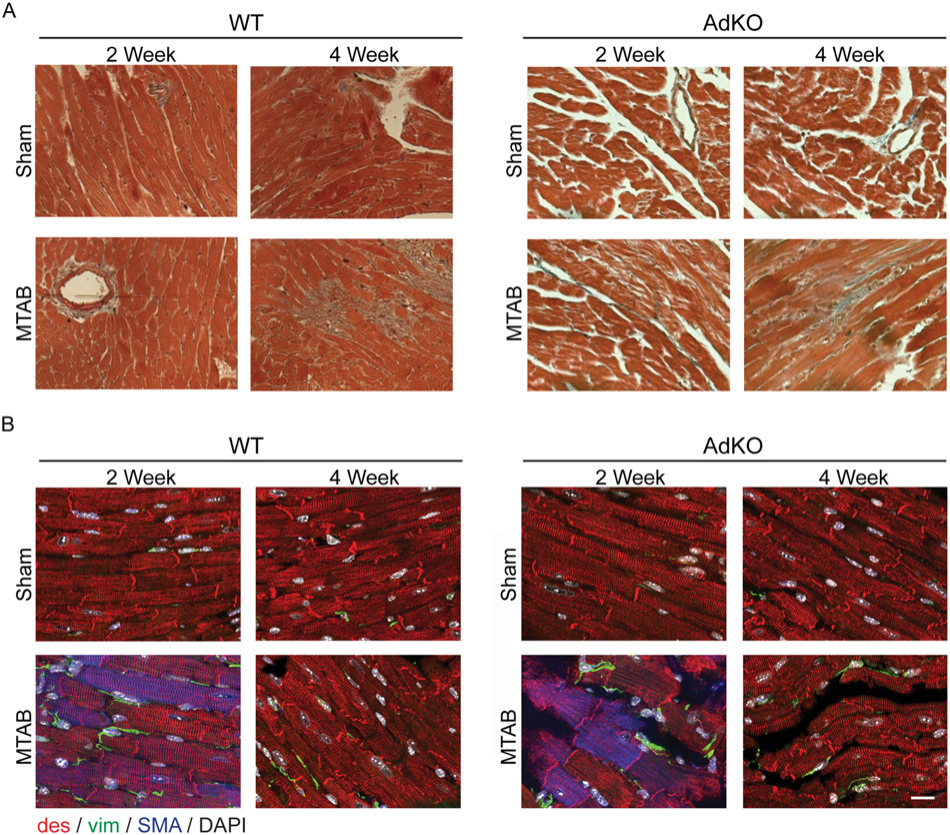
Effects of MTAB in WT and AdKO animals on myocardial cells. (A) Masson’s trichrome staining of histological sections taken from Ad KO or WT mice after 2 or 4 weeks following sham or MTAB surgery. Images shown are representative of 5–10 images of n = 4 to 6 mice per group. (B) WT and AdKO (KO) mice were Sham operated or subject to aortic banding (MTAB) and sacrificed after 2 and 4 weeks post PO. Transmural blocks of the left ventricular myocardium were sectioned and immunostained for α-SMA (SMA, blue), desmin (des, red), vimentin (vim, green) and nuclei (DAPI, white). Scale bar: 50 μm.

**Fig 5:**
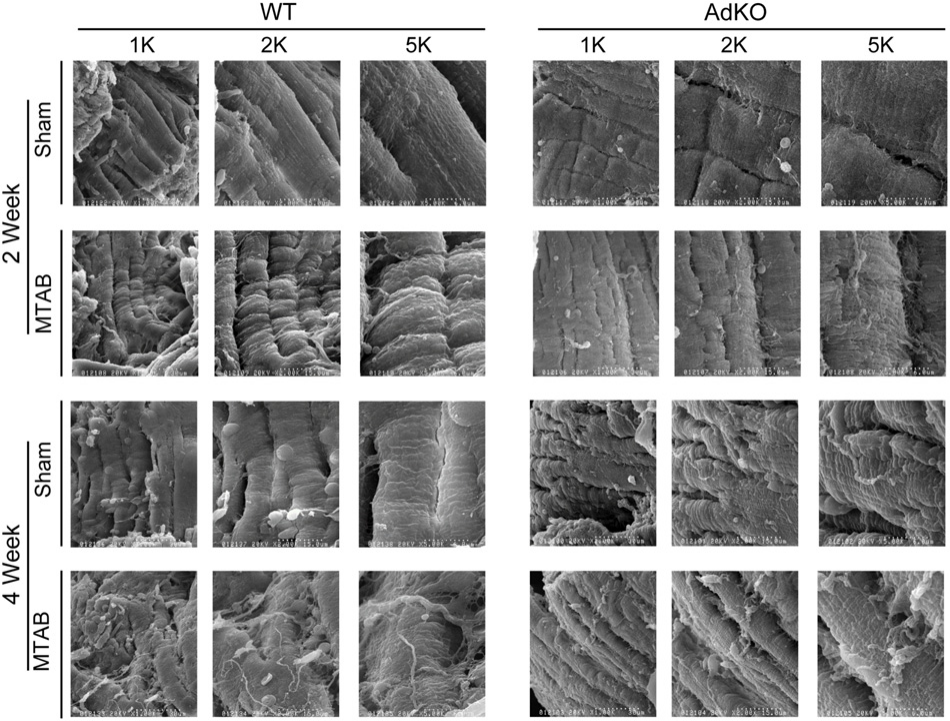
Adiponectin deficiency is associated with increased basal cardiac fibrosis. Representative scanning electron micrographs of fixed left ventricular samples from Ad KO or wt C57BL/6 mice 2 or 4 weeks following sham or MTAB surgery. Magnifications shown are x1K, 2K or 5K as indicated. Images shown are representative of 5–10 images of n = 4 to 6 mice per group.

To examine the changes in myocardial ECM occurring after PO in an adiponectin-deficient background we first performed analysis of total myocardial collagen accumulation using Masson’s Trichrome staining. This also demonstrated an increase in response to 2 and 4 weeks of PO in WT mice, while fibrosis in AdKO mice was notably absent after 2 weeks but highly apparent after 4 weeks of PO (Fig 4A). Surprisingly, despite a generally compromised structure in the AdKO myocardium, time and intensity of fibroblast appearance after MTAB was not significantly different between AdKO and WT animals (Fig 4B). Detailed analysis of 3-dimensional collagen structures using scanning electron microscopy in WT mice, as in Fig 1, revealed accumulation of disorganized small fibres after 2 weeks of PO and large fibre fibrosis after 4 weeks whereas in AdKO mice there was no obvious difference after 2 weeks and a similar phenotype as observed in WT mice after 4 weeks (Fig 5). Again, no apparent changes occurred in sham operated animals of either genotype (Fig 5).

**Fig 6:**
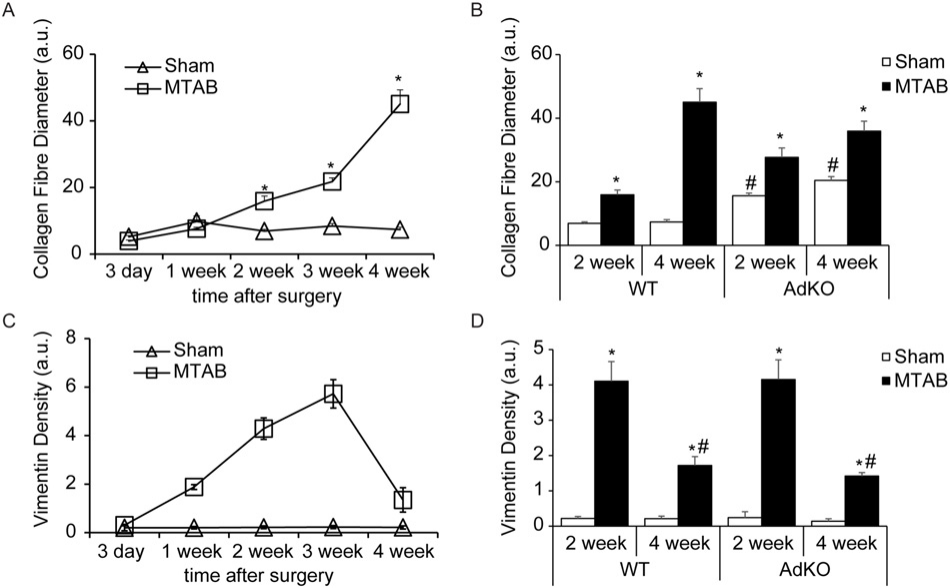
Quantitative analysis of scanning electron microscopy and histology studies. Quantitative analysis of collagen fibre diameter (A&B) and vimentin staining (C&D) was assessed as described in methods. (A&B) Five images were taken per mounted tissue and three tissue samples were produced per animal heart (= 15 images/animal heart). (C&D) Five images were taken per stained slide and three slides were produced per animal heart (= 15 images/animal heart). Mean values and standard deviations were calculated form the values obtained for three different animals per experimental condition (n = 3) and values are mean ± SEM.

Quantitative analysis of scanning electron micrographs showed a progressive increase in collagen fibre diameter following PO surgery (Fig 6A). Basal collagen fibre diameter was higher in KO sham mice compared to WT sham mice, while the thickening of collagen fibres following PO was more pronounced in WT mice compared to KO mice (Fig 6B). Similarly, quantification of vimentin density from confocal images (Fig 3) showed a progressive increase in vimentin until 3 weeks following PO. Vimentin density was restored to near basal levels at four weeks after PO (Fig 6C). Genotype did not affect changes in vimentin expression following PO (Fig 6D).

**Fig 7:**
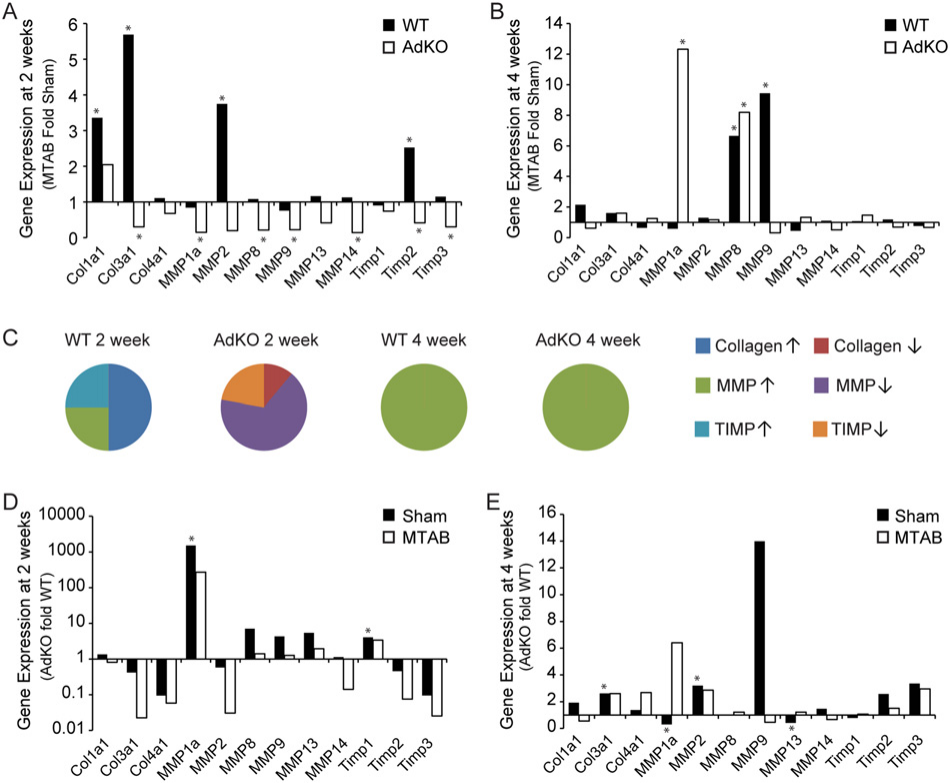
Regulation of ECM-related gene expressions following PO. Analysis of myocardial collagen, MMP, and TIMP expression from Ad KO or wt C57BL/6 mice 2 or 4 weeks following MTAB surgery. (A&B) Quantification of PO fold over sham changes in myocardial mRNA expressions represented as average C(t) value fold sham, where sham is set to 1. (C) Graphical representation of fold over sham mRNA change of collagens (up: blue, down: brown), MMPs (up: green, down: purple), and TIMPs (up: teal, down: orange). (C&D) Quantification of AdKO versus wt changes in myocardial mRNA expressions after PO represented as average C(t) value fold sham, where sham is set to 1. All values are average of n = 4 to 6 mice per group. * = p < 0.05.

We next examined changes in myocardial gene expression of collagen, MMP, and TIMP isoforms and observed a notably different profile in these groups of genes after 2 and 4 weeks of PO and depending on genotype. After 2 weeks of PO, significantly increased levels of Col1α1 and Col3α1, MMP2 and TIMP2 were observed, but these were absent in AdKO mice (Fig 7A). Instead, in AdKO mice numerous gene expressions were reduced (Fig 7A). At 4 weeks the overall changes in gene expressions were less pronounced although MMP8 was elevated in both genotypes, MMP9 in wt mice and MMP1a only in AdKO mice (Fig 7B). The fact that matrix-related gene expression changes were most dramatic at 2 weeks after PO is evident from pie charts showing overall classes of gene expression changes (Fig 7C). To allow easy direct visual analysis of genetype effect we also show the changes in matrix-related genes at 2 and 4 weeks after PO (Fig 7D and 7E, respectively).

### Adiponectin stimulates cardiac fibroblast activity and inhibits angiotensin II (Ang II) induced fibroblast to myofibroblast differentiation

**Fig 8:**
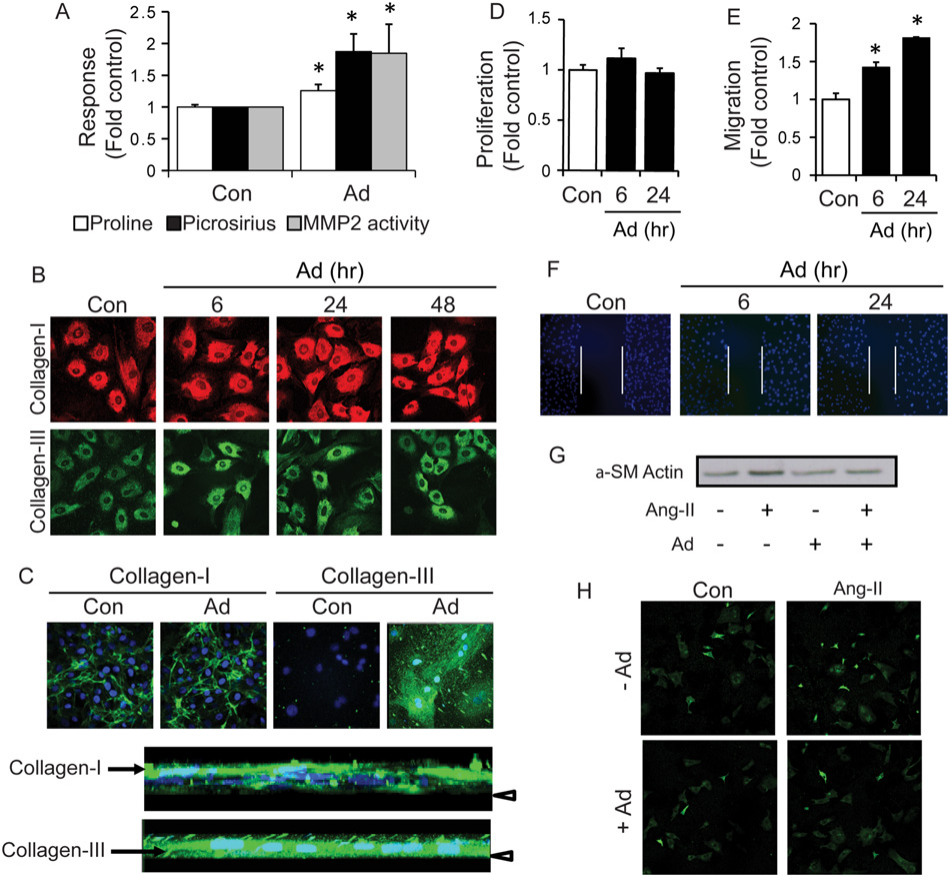
Adiponectin increases collagen synthesis and secretion, and MMP2 activation from cardiac fibroblasts. Adiponectin stimulation of isolated neonatal cardiac fibroblasts was followed by analysis of: (A) Intracellular pro-collagen synthesis was assessed by ^3^H-proline incorporation following adiponectin treatment (5 μg/mL) for 6, 24 or 48 h. Data represent mean values ± SEM from n = 3 experiments using 3 wells per group for quantification. Total secreted collagen was measured in fibroblast conditioned media following adiponectin treatment for 6, 24 or 48 h by picrosirius red staining. MMP2 activation was analyzed by gelatin zymography in conditioned media collected from 6, 24 and 48 h adiponectin treated fibroblasts. Data represented as mean arbitrary units ± SEM from n = 7 experiments. (B) Immunofluorescent images of intracellular collagen I (red) and collagen III (green) synthesized in cardiac fibroblasts at 60x magnification. Cells were treated with adiponectin for 6, 24 and 48 h. Representative images from n = 3 experiments are shown. (C) Immunofluorescent images of extracellular collagen I (green) and collagen III (red) secreted from cardiac fibroblasts at 60x magnification. Cells were treated with adiponectin for 3 days. Cell nuclei were also stained with DAPI (blue). Representative images from n = 3 experiments are shown. Below, 3-dimensional stacks of Ad-treated NCFs immunostained for Collagen I and Collagen III were rotated to show relative vertical orientation of nuclei (DAPI) and collagen (green). Arrow head indicates coverslip. (D) Fibroblast proliferation was assessed by ^3^H-thymidine incorporation following adiponectin treatment for 6, 24, or 48 h. Data represent mean values ± SEM from n = 3 experiments using 3 wells per group for quantification. (E) Fibroblast migration was assessed using the wound-scratch assay following adiponectin treatment for 6 or 24 h. Cell nuclei were stained with DAPI and imaged using confocal microscopy under a 20x objective. Data represent mean values ± SEM from n = 3 experiments using 7–10 images per group for quantification. Representative images are shown in (F). (G) Western blot analysis of cardiac fibroblast cell lysates treated with adiponectin (5 μg/mL) and/or pre-treated with AngII (1 μM). (H) Immunofluorescent staining for αSMA in cardiac fibroblasts treated with adiponectin and/or pre-treated with AngII (1 μM), and imaged using confocal microscopy under a 20x objective.

To this end, our *in vivo* results suggest that myocardial adiponectin affects the remodeling activity but not number of myocardial fibroblasts in conditions of PO. We next used primary CFs to determine the direct effects of adiponectin on these cells *in vitro*. Adiponectin treatment of CFs significantly increased collagen synthesis, examined by 3H-proline incorporation, and secretion, measured by picrosirius staining (Fig 8A). This result was further supported by increased immunofluorescence detection of intracellular collagens type I and III following adiponectin treatment (Fig 8B). Immunostaining of extracellular collagens I and III also highlighted increased accumulation of these collagens in response to adiponectin treatment (Fig 8C). Rotation of stacked confocal images indicated localization of the collagen I ECM above CF monolayers, whereas collagen III appeared as thick vertical fibres between CFs (Fig 8C). Analysis of conditioned media using gelatin zymography showed that adiponectin also stimulated an increase in the active form of MMP2 (Fig 8A). CF proliferation measured by 3H-thymidine incorporation was not altered by adiponectin (Fig 8D); however, addition of adiponectin significantly increased CF migration measured using a wound scratch assay (Fig 8E and 8F). We used Ang II as a positive control to induce fibroblast to myofibroblast differentiation, confirmed by increased expression of α-SMA via Western blot analysis (Fig 8G) and immunofluorescence staining (Fig 8H) and observed that adiponectin pre-treatment significantly attenuated Ang II-induced fibroblast-to-myofibroblast differentiation (Fig 8G and 8H).

Early studies using AdKO mice demonstrated exaggerated cardiac remodelling following induced PO and are often cited as evidence of a cardioprotective effect of adiponectin [6,8,30]. However, emerging clinical data and animal model studies now highlight the more complex association of adiponectin with various stages of cardiac remodeling and progression of heart failure [12–14]. Indeed, a permissive role has been suggested for adiponectin in late stage adverse remodelling events [10,11]. While it remains undisputed that adiponectin plays an important role in the response of the heart to PO [31], when and precisely how adiponectin confers cardioprotective or maladaptive effects must be further clarified. Therefore, we performed a detailed analysis of the temporal progression of cardiac fibrosis induced by PO in the presence or absence of adiponectin *in vivo*, and to examine mechanisms for the direct effects of adiponectin *in vitro*.

The myocardial fibrotic response to PO initially involves an increase in the collagen-I:collagen-III ratio that contributes to improved support for the overloaded myocardium [32,33]. Whereas previous studies have principally relied on Masson’s trichrome staining as a measure of fibrosis [34], we conducted 3-dimensional analysis of developing myocardial fibrosis using scanning electron microscopy. In our study of the progression of fibrosis, PO in WT mice caused an acute increase in small-fibre fibrosis 3 days after surgery, morphologically consistent with the increased deposition of collagen-III, followed by the appearance of thicker collagen-I fibres, beginning after 2 weeks of PO.

At the genomic level, 2 weeks of PO in WT mice elevated collagen-I and -III as well as MMP2 expression in WT mice but failed to induce these changes in the AdKO myocardium. Instead, we observed a widespread downregulation of MMP and TIMP gene expression. One possible interpretation of these data is that adiponectin is required for MMP2 expression in this model. Indeed, our *in vitro* zymography analysis of MMP activity using primary CFs demonstrated a greater level of MMP2 activity after adiponectin. Although the regulation of MMP activity by adiponectin may have been underappreciated to date, previous work has also shown that globular adiponectin stimulated MMP2 in CFs [21] and that adiponectin-stimulated trophoblast invasion occurred at least in part as a consequence of MMP2 activation [35]. The progressive nature of PO-induced myocardial ECM remodeling was highlighted in our study since even though clear differences were apparent after 2 weeks, the genomic changes in WT and AdKO mice were similar after 4 weeks of PO. This is in keeping with functional echocardiography analysis (data not shown) which also indicated that the WT and AdKO mice became more similar after 4 weeks of PO.

We have previously suggested that regulation of cardiac fibrosis may contribute to cardioprotective effects of adiponectin and it was recently suggested that adiponectin has therapeutic implications in the prevention of progression of diabetic nephropathy by antagonizing detrimental effects of Ang II [36]. Others have shown that globular adiponectin enhanced Ang II-induced collagen synthesis [37] and most intriguingly, a recent study suggests that adiponectin reduced AngII-induced cardiac fibrosis via activation of autophagy in macrophages [38]. Our present in vitro studies in primary fibroblasts supports that adiponectin directly enhances collagen synthesis, measured by proline incorporation and picrosirius red assays. This was also reflected in immunofluorescence analysis of collagen types I and III, with the change in the latter being the most striking observation as rod-like extracellular structures were evident in z-plane view.

We also performed a histological examination of the cellular composition of the myocardium and in particular the myofibroblast content [39]. The presence of α-SMA positive cells in the myocardium is a common indicator of myocardial stress and decreased ventricular compliance [15], while localization of differentiated myofibroblasts to the peri-infarct area has been associated with decreased cardiac function following myocardial infarct, as myofibroblasts secrete high levels of MMPs and collagen as part of the innate cardiac repair mechanism [40]. In our model of MTAB, we did not observe differentiation of myocardial fibroblasts into differentiated α-SMA expressing myofibroblasts but clear activation of vimentin-positive fibroblastic cells in the myocardial interstitium. The absence of α-SMA staining in these cells suggests that the PO response in our *in vivo* our model was mostly driven by proto-myofibroblasts that are characterized by ECM production and remodeling rather than pathological contraction of ECM into scar tissue [16]. It is likely that the increase of interstitial fibroblasts during the first 2 weeks after MTAB accounted for the structural changes observed in the collagenous ECM. Stagnation and decrease of fibroblast content after 2 weeks is indicative of the completion of the adaptive response to PO. Typically, immediate expansion of the ECM following PO and MI is associated with adaptive remodelling, contributing to the preservation of cardiac function. Here we observed that ECM fibrosis following 4 weeks of MTAB was reduced in AdKO mice compared to WT mice suggesting a delay in ECM remodelling due to adiponectin deficiency. This difference in ECM fibrosis was not accompanied by substantially different accumulation of fibroblasts in the myocardial interstitium. Our in vitro results suggest that adiponectin controls the ECM secreting and remodelling activity of cardiac fibroblasts rather than their numbers. Taken together, we postulate that adiponectin deficiency permits the presence of activated fibroblasts in the normal myocardium, thereby inducing non-pathological small fibre cardiac fibrosis that confers short-term protection of cardiac function following the induction of PO. The contribution of cardiomyocytes in the structural ECM alterations following PO remains elusive. However, we observed markedly increased numbers of cardiomyocytes expressing increasing levels of α-SMA during the first 2 weeks following PO in WT and AdKO animals. Cardiomyocyte expression of α-skeletal actin and α-SMA has been described previously in experimental rodent and pathological human heart following pressure overload and hypertension [41–45]. Expression of these actin isoforms that appear evolutionary and developmentally earlier than α-cardiac actin is indicative of the return to an embryonic gene expression program characteristic for cardiac tissue under repair and remodelling [46,47].

To study whether adiponectin directly influenced fibroblast activation, we treated primary CFs with AngII to induce differentiation to myofibroblasts [48] and observed that adiponectin pre-treatment significantly inhibited Ang II-induced CF differentiation. In conventional culture conditions, fibroblast spontaneously differentiate into proto-myofibroblasts and expression of α-SMA in these is the next differentiation step [16]. Indeed, there is likely great pathophysiological significance in the crosstalk between adiponectin and Ang II actions [49]. Moreover, our data is in keeping with previous work which has shown that adiponectin inhibited lipopolysaccharide-induced adventitial fibroblast transition to myofibroblasts [50] and inhibited α-SMA gene expression in dermal fibroblasts [51]. Adiponectin has also been shown to attenuate other effects mediated by AngII, such as oxidative stress, inflammation and atherosclerosis [52,53]. Furthermore, cardiac fibrosis induced by AngII-infusion was more severe in AdKO compared to WT mice and could be reduced by adenoviral-mediated adiponectin replenishment [54]. Fibroblast migration is also an important component of cardiac remodeling and previous studies have shown that the globular C-terminal form of adiponectin increased basal and enhanced AngII-induced proliferation of cardiac fibroblasts [21,37]. Here we show that full length adiponectin directly promoted CF migration without affecting proliferation. Thus, it is now clear that regulation of CF function in both basal and pathological settings is an important facet of adiponectins influence on cardiac function.

The question remains whether the differences observed in AdKO versus WT animal hearts under PO are due to lack of circulating or local adiponectin. It becomes increasingly accepted that adiponectin not only exerts endocrine effects but that autocrine and paracrine effects of adiponectin produced by various tissues, including the heart, are physiologically important [5,55]. We investigated cardiac adiponectin mRNA and protein content in the current study and contrasted this with circulating levels. We found that after PO, particularly at 4-weeks, there was enhanced adiponectin protein concentration in cardiac homogenates. Interestingly, this occurred despite reduced myocardial adiponectin mRNA levels and a slight but significant decrease in circulating total adiponectin levels. One possible interpretation of this data is that there was increased retention of adiponectin within the fibrotic myocardium and this could occur due to enhanced collagen content. Indeed, it was proposed that adiponectin itself acts as a scaffold of newly formed collagen in myocardial remodeling [56]. Another factor dictating the myocardial actions of adiponectin is the transport from circulation to interstitial space [57,58] and it may be that endothelial transport of adiponectin is altered in the overloaded hypertrophied heart. The importance of enhanced bioavailability via production or accumulation adds to the complexity of understanding adiponectin action in the heart.

## Conclusions

In conclusion, using the model of WT or AdKO mice with mild PO, our data indicate that adiponectin deficiency delays the progression of PO induced fibrosis. Small fibres in the AdKO myocardium that serve to support the heart against hemodynamic load may be a result of AngII-induced myofibroblast differentiation permitted by adiponectin deficiency. In WT mice, however, ECM expansion following PO may serve to retain adiponectin within the myocardium. These data further add to our understanding of the role played by adiponectin in cardiac remodeling and highlight the important temporal nature of such effects.
